# Plant-Derived Nanovesicles: A Novel Form of Nanomedicine

**DOI:** 10.3389/fbioe.2020.584391

**Published:** 2020-10-14

**Authors:** Lanlan Yu, Zhun Deng, Lei Liu, Wenbo Zhang, Chenxuan Wang

**Affiliations:** ^1^State Key Laboratory of Medical Molecular Biology, Institute of Basic Medical Sciences, Chinese Academy of Medical Sciences and Peking Union Medical College, Beijing, China; ^2^Department of Chemistry, University of Wisconsin-Madison, Madison, WI, United States

**Keywords:** plant-derived nanovesicles, cross-kingdom gene regulation, nanomedicine, bioeffects of nanomaterials, small RNAs

## Abstract

The nanovesicles extracted from the plant and herbal decoctions are identified as a new class of nanomedicine. They are involved in interspecies chemical communication across the plant and animal kingdoms and display a therapeutic potential against a variety of diseases. Herein, we review the recent progress made in the medical applications of plant-derived nanovesicles in the aspects of anti-inflammation, anti-cancer, tissue regeneration, and modulating commensal microbiota. We further summarize the cellular and molecular mechanisms underlying the physiological functions of plant-derived nanovesicles. Overall, plant-derived nanovesicles provide an alternative to conventional synthetic drugs and present exciting opportunities for future research on disease therapy.

## Introduction

Plant-derived nanovesicles are a group of nano-scaled vesicles that are isolated from dietary vegetables and fruits (Zhang et al., 2016b; Yang et al., 2018a; Li et al., 2019). These nanovesicles contain a broad range of membrane-bounded structures from distinct origins, including (1) natural extracellular vesicles (including exosomes) as well as intracellular vesicles existing in plants; (2) artificial vesicles formed during the preparation and extraction process; (3) synthetic liposomes or nanovectors prepared from plant-derived molecules as natural vesicle mimics. The chemical compositions of plant-derived nanovesicles include lipids, proteins, nucleic acids, and secondary metabolites, etc. Plant-derived nanovesicles possess high biocompatibility and promise a large-scale production. More and more evidences suggest that plant-derived nanovesicles can enter mammalian cells and mediate plant-animal cross-kingdom gene regulation, i.e., plant small RNAs packed by plant-derived nanovesicles could survive in the active form in animals and exogenously modulate the host cellular processes via genetic crosstalk (Li et al., 2019). It indicates a potential medical application of plant-derived nanovesicles in the regulation of the fundamental biological processes in the human body. Currently, plant-derived nanovesicles have exhibited promising activities in the homeostatic regulation of the immune system, development of tissue engineering and reconstruction, delivery of chemotherapeutic drugs and nucleic acids, etc. (Ju et al., 2013; Quesenberry et al., 2015; Zhang et al., 2016a,b,c; Yang et al., 2018a; Li et al., 2019). In addition, compared to the currently available drug delivery systems, plant-derived nanovesicles have multiple advantages, such as low immunogenicity and stability in the gastrointestinal tract (Yang et al., 2018a).

In this review, we outline the recent achievements made in the therapeutic applications of plant-derived nanovesicles in respect of anti-inflammation, anti-cancer, tissue regeneration, and modulating commensal microbiota. The molecular and cellular mechanisms responsible for the bio-functions conferred by plant-derived nanovesicles are also discussed. We hope this review conveys the main concept that plant-derived nanovesicles represent a combinatory approach in precision medicine and emerge as the next generation of nanomedicine with multi-functionalities.

## Application in Inflammation Regulation

**FIGURE 1:**
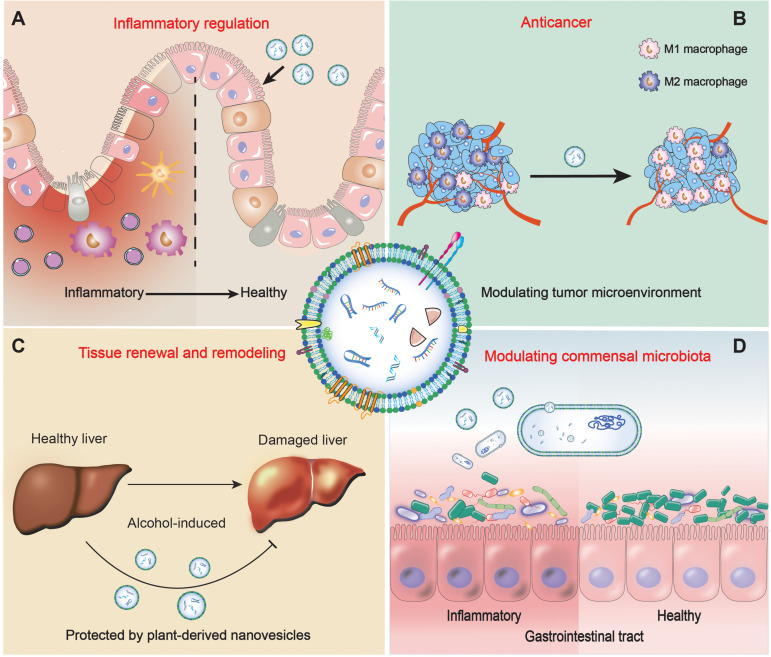
Schematic representations of medical applications of plant-derived nanovesicles **(A)** in inflammation regulation, **(B)** in anticancer, **(C)** in tissue renewal and remodeling, **(D)** in modulating commensal microbiota.

Plant-derived nanovesicles are proved to play a critical role in maintaining intestinal immune homeostasis through the communication with the intestinal cells. Ulcerative colitis (UC) is a major type of inflammatory bowel disease. Conventional steroidal drugs and immunosuppressants exhibit poor therapeutic effects due to the non-specific targeting to the pathogenesis and an unavoidable toxicity to the normal cells (Wang et al., 2014). Therefore, the development of non-toxic delivery systems that target colonic tissues and have high anti-inflammatory properties is essential for the treatment of UC. Grapefruit-derived nanovesicles (size: 211 ± 49 nm) extracted by sucrose gradient have been reported to be selectively taken up by intestinal macrophages and to ameliorate dextran sulfate sodium (DSS)-induced colitis in mice (Wang et al., 2014; Figure 1A). The anti-inflammatory effects of the nanovesicles were mediated through upregulating the expression of heme oxygenase-1 and downregulating the production of IL-1β and TNF-α in the intestinal macrophages. Additionally, grapefruit nanovesicles exhibit inherent biocompatibility, biodegradability and stability over a wide range of pH values. These properties endow grapefruit nanovesicles with a capacity to function as a delicate designed oral delivery system to deliver anti-inflammatory drugs, such as methotrexate (MTX), to lower the cytotoxicity and improve the therapeutic effect. An alternative commonly used plant-derived nanovesicles are ginger-derived nanoparticles (size: 233 nm), which were isolated from homogenized ginger and then purified by sucrose gradient centrifugation. Then total lipids were extracted from ginger nanoparticles by using the Bligh and Dyer method to develop a novel siRNA delivery system for UC therapy (Zhang et al., 2017). After the loading of the siRNA against *cd98* (a colitis-induced gene with up-regulated expression), these functionalized liposomes (size: 190 nm) could specifically target the intestinal epithelial cells and deliver CD98 siRNA to reduce colonic *cd98* gene expression. In addition, the colon immune system can also be regulated by plant-derived nanovesicles through modulating the enzyme reaction pathways. For example, broccoli-derived nanoparticles (size: 32 nm), extracted from broccoli juice by centrifugation and isolated using a pressure-regulated pump into a Biomax-500 column, can inhibit mouse colitis by activating adenosine monophosphate-activated protein kinase (AMPK) to prevent the activation of dendritic cell in intestine (Deng et al., 2017).

Another way to modulate the innate immune response is to regulate the activation of particular inflammasomes (Broz and Dixit, 2016; Ding et al., 2019; Spel and Martinon, 2020). Among various types of inflammasomes, nucleotide-binding domain and leucine-rich-repeat-containing family and pyrin domain-containing 3 (NLRP3) is implicated in Alzheimer’s disease and type 2 diabetes upon aberrant activation. A medicine with the potential to inhibit the activation of NLRP3 inflammasomes is required to combat these diseases. The nanovesicles that are extracted from ginger rhizomes via a centrifugation and sucrose gradient purification show inhibitory effects on the NLRP3 inflammasome activation in the primary macrophages (Chen et al., 2019). The administration of ginger rhizomes-derived nanoparticles suppressed the downstream signaling pathways of inflammasome activation, including caspase1 autocleavage, interleukin (IL)-1β and IL-18 secretion, and pyroptotic cell death. The molecular mechanism underlying the inhibitory activity mediated by the ginger rhizomes-derived nanoparticles is attributed to the pivotal role of the lipid components of nanoparticles to block the assembly of NLRP3 inflammasomes.

## Application in Anticancer

Plant-derived nanovesicles exhibit a set of promising activities that can be used in the medical therapy against cancer in four aspects: selective apoptosis activation of tumor cells, regulation of inflammatory factors, modulation of tumor microenvironment, and delivery of therapeutic agents. (1) Selective apoptosis activation of tumor cells. The tumor necrosis factor-related apoptosis-inducing ligand (TRAIL) is a key target that selectively induces the apoptosis of cancer cells without affecting normal cells. Plant-derived nanovesicles exhibit an activity to activate the TRAIL signaling. For example, Raimondo et al. showed that citrus limon-derived nanovesicles (size: 50–70 nm) can inhibit the proliferation of different types of solid and hematological cancers cells *in vitro* and suppress chronic myeloid leukemia xenograft tumor growth *in vivo* (Raimondo et al., 2015). The selectively increased level of TRAIL and its receptor Dr5 was observed in cancer cell lines, indicating that an autocrine loop induced by citrus limon-derived nanovesicles is responsible for the apoptosis of cancer cells. For the cancer cells, a dose of 20 μg/ml of nanovesicles induced 50% growth reductions at 48 h. In the tumor xenograft model, at day 21 post-implantation, tumor weight in the treated mice were about 15–30% compared with control. (2) Regulation of inflammatory factors. Zhang et al. (2016a) found that edible ginger-derived nanoparticles (size: 232 nm) could prevent colitis-associated cancer. By orally administered ginger nanoparticles, the internal mRNA level of pro-inflammatory cytokines IL-6, IL-1β, and the proliferation-related cyclin D1 substantially decreased, parallel with a decrease in the tumor numbers and tumor loads. This result reveals that ginger-derived nanoparticles suppress colorectal tumorigenesis by reducing pro-inflammatory cytokine levels and mediating intestinal epithelial cells metabolism. (3) Modulation of tumor microenvironment. Tumor-associated macrophages (TAMs) are a kind of the major components in the tumor microenvironment. TAMs are considerably plastic and can be polarized into two opposite phenotypes, the tumoricidal M1 and tumor-supportive M2 macrophages. Most types of tumors are associated with a lower ratio of M1/M2 (Biswas and Mantovani, 2010; Goswami et al., 2017). Stimulating the polarization of TAMs to M1 phenotype can be beneficial to inhibit the growth of cancer cells. *In vivo* experiments proved that ginseng-derived nanoparticles (size: 345 nm) can effectively inhibit melanoma growth by altering the macrophage polarization (Cao et al., 2019) (Figure 1B). Relative to the control group, the ratio of M1/M2 of B16F10-allografted mice treated by ginseng-derived nanoparticles was significantly improved at day 12. The tumor weight of treated mice was reduced by 53%, and the treated mice gained more body weight than the control group. Suppressing the M2-like polarization of macrophages is mediated by the specific ligands carried by ginseng-derived nanoparticles to interact with Toll-like receptor 4 (TLR4) and a consequent activation of myeloid differentiation antigen 88 (MyD88)-dependent pathway. (4) Therapeutic agent delivery system. Lipids extracted with the Bligh and Dyer method from plant-derived nanovesicles can load antitumor agents (including small molecular drugs, nucleic acids, and proteins etc.) as nanovectors for cancer therapy. For instance, the grapefruit-derived bioengineered lipids facilitate the delivery of miRNA drugs (miR-18a as a tumor suppressor) to inhibit liver metastasis through induction of M1 macrophages (Teng et al., 2016).

## Application in Tissue Renewal and Remodeling

The human gut is exposed on a daily basis to billions of nanovesicles from regular diet. The edible plant-derived nanovesicles have profound impacts on intestinal tract, i.e., intestinal tissue renewal and remodeling (Ju et al., 2013; Mu et al., 2014). For instance, grape-derived nanoparticles (size: 381 ± 37 nm), isolated by differential centrifugation and sucrose gradient ultracentrifugation, were reported to promote intestinal stem cell proliferation (Ju et al., 2013). Treated with grape nanovesicles, a number of genes including *axin-2*, *cyclin D1*, *c-myc* and *egfr*, which regulate intestinal stem cell growth, were significantly up-regulated through the Wnt/β-catenin signaling pathway in the stem cells. Alternatively, blocking β-catenin-mediated signaling pathways attenuated the production of stem cells. Further evidence showed that grape-derived nanovesicles can directly promote the proliferation of intestinal stem cells and accelerate the formation of crypt organoids from a single isolated stem cell. In addition, orally administrated grape nanovesicles can also protect mice against DSS-induced colitis via inducing intestinal stem cells. Therefore, grape nanovesicles exhibit the capacity of both modulating intestinal tissue renewal processes and participating in the remodeling of it in response to pathological triggers.

Another widely investigated plant-derived nanovesicles, ginger-derived nanoparticles (size: 387 nm) isolated by using a sucrose gradient centrifugation method, were demonstrated to protect mice against alcohol-induced liver damage (Zhuang et al., 2015; Figure 1C). Treated with ginger nanoparticles, primary hepatocytes had a significantly increased nuclear translocation of nuclear factor erythroid 2-related factor 2 (Nrf2), which transcriptionally controls the gene expression of many cytoprotective enzymes. In detail, the activation of Nrf2 mediated by ginger nanoparticles resulted in an induction of serious liver detoxifying/antioxidant genes including *ho-1*, *nqo1*, *gclm*, and *gclc*, and an eliminated production of reactive oxygen species (ROS), which benefits to protect liver against insults. Additionally, it has been further identified that the key component is the shogaol lipid in the ginger-derived nanoparticles, which affects the induction of Nrf2 in a TLR4/TRIF-dependent way. This research not only demonstrates that ginger nanoparticles could be used as a novel nanomedicine to protect the liver against alcohol-induced damage but also provides a foundation for unveiling the molecular mechanism underlying interspecies communications mediated by different kinds of edible plant nanovesicles we ingest daily.

Notably, a special class of plants, herbs have also been investigated in the aspects of tissue repair and remodeling. Herbs, consumed by drinking decoctions, have been used as therapeutic medicine for thousands of years. Decoctions obtained by boiling herbs with water contain many functional and heat-stable components (Yang et al., 2016; Ji et al., 2019). Different from the natural extracellular vesicles and intracellular vesicles identified in plants, the nanovesicles derived from the decoctions are artificially generated during decoctions and infusions. For example, the decoction of Hong Jing Tian (HJT, RHODIOLAE CRENULATAE RADIX ET RHIZOMA, *Rhodiola crenulata*), can ameliorate the activation of TGF-β1-induced MRC-5 fibrotic cells and the pulmonary fibrosis in the bleomycin-induced mice (Du et al., 2019). Subsequently, Li et al. defined the heat-stable nanovesicles from HJT decoctions as *decoctosomes* (size: 198 nm) and reported that they possess more potent therapeutic effects *in vitro* and *in vivo* than the corresponding decoctions (Li et al., 2019). A component analysis of HJT decoctosomes demonstrated that the specific sRNA HJT-sRNA-m7 exhibits potent anti-fibrosis effects. Additionally, the simplified mimic of decoctosome is defined as *bencaosome*. The simplified form was artificially synthesized by mixing and heating the lipid sphinganine (d22:0) and its functional sRNA, which are both identified from the components of decoctosome. Following oral administration in mice, sphinganine-HJT-sRNA-m7 bencaosomes effectively ameliorate bleomycin-induced lung fibrosis. The differences in boiling temperature, decoction duration, and the raw materials employed may produce the resulting liquids with different chemical and biological properties. Consequently, the physiochemical properties and biological activities of herb-derived nanovesicles are significantly affected by the preparation/decoction process.

## Application in Modulating Commensal Microbiota

Commensal microbiota has been shaped by co-evolution with the human host. The number of bacterial cells in a healthy adult human body is about 100 trillion and 10 times as many as human cells. The situations of commensal microbiota are closely related to human health for participating in various physiological activities, ranging from food degradation to immune regulation. The dysbiosis of the commensal microbiome causes numerous health problems. Over the last few decades, myriad studies have demonstrated the relationship between diet and microbiota, especially for the gut microbiota. Edible plant, including herbs, can regulate the composition of the commensal microbiome (Chen et al., 2016; Li et al., 2017).

Recent research work has shown that plant nanovesicles are stable in the gut and can be taken up by commensal bacterium, leading to changes in gut microbiota and affecting host. For example, Teng et al. found that gut bacteria *Lactobacillus rhamnosus* (LGG) can be regulated by the sRNA from ginger nanovesicles via binding with bacterial mRNA (Teng et al., 2018; Figure 1D). Ginger-derived nanovesicles (size: 207 ± 81 nm) were preferentially taken up by LGG in a lipid-dependent manner. *In vivo* experiments showed that after the oral administration of ginger nanovesicles, the growth of LGG was promoted. The mechanism is mainly attributed to the small RNAs carried by ginger-derived nanovesicles binding to various genes of LGG. For example, gma-miR396e targets LGG transcription repressor LexA mRNA, lowering the expression level of *lexA*. Mdo-miR7267-3p targets LGG *ycnE* gene, suppressing monooxygenase and enhancing indole-3-carboxaldehyde (I3A). Excess I3A induces the secretion of IL-22, which enhances gut barrier function and ameliorates mouse colitis. And miR167a-5p targets LGG pilus-specific protein SpaC gene that is responsible for the bacterial migration. With a less expression of SpaC proteins on pili, the abilities of LGG invading into gut epithelial cells and migrating into the bloodstream and liver are reduced.

Another research work demonstrated ginger-derived nanovesicles (size: 204 nm) protect oral environment by inhibiting the pathogenicity of *Porphyromonas gingivalis* (*P. gingivalis*) (Sundaram et al., 2019). *In vivo* experiments demonstrate that the treatment with ginger-derived nanovesicles significantly decreases *P. gingivalis*-induced alveolar bone loss and the release of inflammation factors. The unsaturated PA (34:2) from ginger-derived nanovesicles facilitates the uptake of nanovesicles by *P. gingivalis* via the interactions between PA (34:2) and the hemin-binding protein 35 on bacterial surface. PA (34:2) and the nanovesicles miRNAs preferentially inhibit the expression of several virulence factors and suppress the attachment and invasion of *P. gingivalis* in oral epithelial cells.

## Conclusion and Future Perspectives

Recent research advances have identified diverse crucial activities of plant-derived nanovesicles in the regulation of immune response, defense, cell differentiation, and proliferation (Ju et al., 2013; Quesenberry et al., 2015; Zhang et al., 2016a,b,c; Yang et al., 2018a; Li et al., 2019). Accumulating evidence reveals that plant-derived nanovesicles can serve as a novel therapeutic agent or therapeutics delivery platform and develop an effective treatment for diseases. Medical plants have been used in traditional medicine practices for thousands of years and offer valuable sources for the discovery of modern pharmaceuticals. The variety and abundance of plant-derived nanovesicles that carry chemicals, proteins, and nucleic acids from traditional herbal medicine may provide far more treatment options than synthetic drugs and protein medicines. In the future, efforts are required in the following directions to promote the medical application of plant-derived nanovesicles.

(1) The identification and analysis of small RNAs derived from plant-derived nanovesicles. In a limited number of innovative studies with the nanovesicles extracted from the decoctions of *Rhodiola crenulata*, *Taraxacum mongolicum*, *Andrographis paniculata*, and *lonicera japonica*, botanic small RNAs are found in mammalian cells and play a crucial role to mediate the functions of plant-derived nanovesicles *in vivo* (Du et al., 2019; Huang et al., 2019; Li et al., 2019). In the next step, a large-scale analysis of the millions of small RNA sequences obtained from the various plant-derived nanovesicles is needed to systematically identify the active small RNAs that potentially regulate host gene expression.
(2) The molecular pathway of plant-derived nanovesicles in the human body. Currently, locating the targets for the active components (small molecules, proteins, nucleic acids) from plant-derived nanovesicles *in vivo* heavily depends on bioinformatics prediction approaches (Du et al., 2019; Huang et al., 2019; Li et al., 2019). There still remain challenges in both of simulation and experiment, affecting the reliability of target determination. It is important to develop a powerful dry lab as well as wet lab approach to gain a better understanding of the pathway of small RNAs in the human body, which can help to understand the biological effects of plant-derived nanovesicles.
(3) Evaluating the biological effects produced by the synthetic mimics of plant-derived nanovesicles. Environmental factors, such as extreme climate events, atmospheric CO_2_ level, soil conditions, etc. can alter plant chemical composition (AbdElgawad et al., 2014; Yang et al., 2018b), which could change the effective composition carried by plant-derived nanovesicles in turn. Some endeavors have been made to reduce the uncontrollable factors in the preparation of plant-derived nanovesicles, such as the synthesis of the simplified and well-defined mimics by using a combination of several active components identified from plant extracts (Li et al., 2019). However, this approach could potentially be inadequate because using only the few known components from the complicated systems is likely to impair the synergic effects of the whole system of biomolecules carried by the plant-derived nanovesicles. In the future, more studies are still needed to compare the biological effects played by the natural nanovesicles versus their simplified mimics.

## Author Contributions

LY, ZD, LL, WZ, and CW discussed and wrote the manuscript. All authors contributed to the article and approved the submitted version.

## Conflict of Interest

The authors declare that the research was conducted in the absence of any commercial or financial relationships that could be construed as a potential conflict of interest.
